# Identification of critical connectors in the directed reaction-centric graphs of microbial metabolic networks

**DOI:** 10.1186/s12859-019-2897-z

**Published:** 2019-06-13

**Authors:** Eun-Youn Kim, Daniel Ashlock, Sung Ho Yoon

**Affiliations:** 1 School of Basic Sciences, Hanbat National University, Daejeon, 34158 Republic of Korea; 2 Department of Mathematics and Statistics, the University of Guelph, Guelph, Ontario N1G 2W1 Canada; 3 Department of Bioscience and Biotechnology, Konkuk University, Seoul, 05029 Republic of Korea

**Keywords:** Directed network, Metabolic network, Reaction-centric graph, Cascade number, Centrality metric, Information flow

## Abstract

**Background:**

Detection of central nodes in asymmetrically directed biological networks depends on centrality metrics quantifying individual nodes’ importance in a network. In topological analyses on metabolic networks, various centrality metrics have been mostly applied to metabolite-centric graphs. However, centrality metrics including those not depending on high connections are largely unexplored for directed reaction-centric graphs.

**Results:**

We applied directed versions of centrality metrics to directed reaction-centric graphs of microbial metabolic networks. To investigate the local role of a node, we developed a novel metric, *cascade number*, considering how many nodes are closed off from information flow when a particular node is removed. High modularity and scale-freeness were found in the directed reaction-centric graphs and betweenness centrality tended to belong to densely connected modules. Cascade number and bridging centrality identified cascade subnetworks controlling local information flow and irreplaceable bridging nodes between functional modules, respectively. Reactions highly ranked with bridging centrality and cascade number tended to be essential, compared to reactions that other central metrics detected.

**Conclusions:**

We demonstrate that cascade number and bridging centrality are useful to identify key reactions controlling local information flow in directed reaction-centric graphs of microbial metabolic networks. Knowledge about the local flow connectivity and connections between local modules will contribute to understand how metabolic pathways are assembled.

**Electronic supplementary material:**

The online version of this article (10.1186/s12859-019-2897-z) contains supplementary material, which is available to authorized users.

## Background

Models and methods from the graph theory have been developed to characterize structural properties in various kinds of complex networks in social, technological, and biological areas [1, 2]. In the analysis of biological networks, graph theory has been successful in detecting global topological features of biological networks such as short path lengths, scale-freeness with the appearance of hubs [3], hierarchical modular structures [4], and network motifs [5]. While the topological analysis as a whole can give insight on network evolution and cellular robustness [3, 6], investigation of influences of individual nodes in a biological network has potential for practical applicability such as identification of drug targets, design of effective strategies for disease treatment [7], and development of microbial hosts for mass-production of various bioproducts [8].

Ranking of a node by its topological feature depends on various centrality metrics, each of which identifies central nodes affecting the network architecture from global or local perspectives [1, 9]. For example, degree centrality and clustering coefficient which are based on nodes’ degree identify nodes of global topological importance of hubs and modules, respectively. Examples of centrality metrics based on information flow are betweenness centrality which is the proportion of shortest paths passing through a node [10] and bridging centrality that identifies bridging nodes lying between modules [11]. Such global topological analyses have been mostly performed using undirected bionetworks. Recent studies extended several global measures, such as in/out-degree distribution, betweenness, closeness, clustering coefficient, and modularity for application into directed networks [1, 12, 13]. These measures are strongly correlated with high degrees, focusing on densely connected sub-structures. Although they discovered global topological properties and global roles of individual nodes, they are insufficient to explain connections between modules and local connectivity, typically within a few of steps of neighbors surrounding the node, in networks with directed flows. For example, nodes of high degree have global topological importance in a network, however, the fact that they have so many interactions means that they are poor channels for conveying information. A signal that controls a specific cellular process must have some specificity in how its signal is received and interpreted [14, 15]. If systems in several parts of the cell responded to the signal, as they do with high degree nodes, the node in question would not be a control for the specific process. Such need for specificity of signal effect means that high degree nodes in the network may be ignored or removed when performing topological analysis to locate nodes that are critical in particular pathways.

As majority of biological networks such as metabolic, gene regulatory, and signal transduction networks show the sequential interaction of elements, they can be best represented as directed graphs [1]. Unlike undirected networks, there is a directed information flow, creating an asymmetric influence between the nodes in a directed network. Any directed path in a network represents a sequence of reactions, ordered in pairs where each is a pre-requisite of the next. Information flow arises from these reaction cascades, and thus, it can represent *the potential for temporal correlation of activity changes in a network.* The information flow through a node in a network can be estimated as the number of nodes downstream from it whose behavior will be influenced if that node is removed or disables. Thus, centrality metrics based on a node’s information flow can be well suited to reflect the directionality of information flow in real biological networks.

Metabolism is the totality of all biochemical reactions that produce building blocks, energy, and redox requirements for cellular functions. Metabolism consists of metabolic pathways, each of which is a directed path from the source metabolites to target metabolites mediated by a sequence of biochemical reactions. Recent sequencing technology and databases of metabolic pathways allow the reconstruction of genome-wide metabolic networks in diverse organisms [16, 17]. Databases about metabolic pathways, such as KEGG [18], Reactome [19], MetaCyc, and BioCyc [20] are available; methods have been developed for the (semi-) automated reconstruction of metabolic networks [21, 22]. The existing availability of databases of metabolic networks has greatly facilitated the computational analysis of metabolic networks.

In general, metabolic networks have been represented as a metabolite-centric graph with the metabolites as nodes and reactions as edges [23–25]. In a metabolite-centric graph, two metabolites are connected if there is a reaction using one metabolite as a substrate and the other as a product. The other way is a reaction-centric graph where two reactions are connected by at least one arc representing a substrate or product metabolite. The practical advantage of the reaction-centric graph is that its topological analysis can yield testable biological insights, such as the identification of essential reactions, which can be experimentally verified by a gene deletion study. Another way to describe metabolic networks is a bipartite graph with two types of nodes representing metabolites and reactions [26], however, centrality metrics used for topological analysis of unipartite metabolic networks cannot be directly applied to the bipartite metabolic graph [13]. So far, centrality metrics for topological analysis of unipartite metabolic networks have been mostly performed with metabolite-centric graphs. Only a few studies have attempted to apply centrality metrics to reaction-centric graphs, such as the topological analysis of cancer metabolic networks using degree-based centrality metrics [13]. Especially, to our knowledge, centrality metrics that are not based on high connections are unexplored for directed reaction-centric graphs.

In this work, we investigated the topological roles of individual reaction nodes in directed reaction-centric graphs using centrality metrics including those not depending on nodes’ degree. We applied various centrality metrics to analysis of directed reaction-centric graphs of metabolic networks of five phylogenetically diverse microorganisms of *Escherichia coli* (Gammaproteobacteria), *Bacillus subtilis* (Firmicutes), *Geobacter metallireducens* (Deltaproteobacteria), *Klebsiella pneumonia* (Gammaproteobacteria), and *Saccharomyces cerevisiae* (Eukaryota). To identify nodes of global topological importance, central metrics depending on high connections (degree, modularity, clustering coefficient, and betweenness centrality) were applied. To investigate the role of a node more locally, we modified bridging centrality reflecting reaction directionality and developed a novel metric called *cascade number*. To link reactions highly ranked with each central metric to their biological importance, the proportions of the essential reactions predicted by flux balance analysis (FBA) were calculated according to the centrality metrics. These analyses identified topological features of individual nodes in the directed reaction-centric graphs from global and local connectivity perspectives.

## Results

We begin by explaining concepts of central metrics using a toy network model. Next, we investigated global features and roles of existing central metrics in the five directed reaction-centric graphs, each of which was derived from the metabolic network model of *E. coli* (iJO1366) [27], *B. subtilis* (iYO844) [28], *G. metallireducens* (iAF987) [29], *K. pneumonia* (iYL1228) [30], or *S. cerevisiae* (iMM904) [31] (Table 1). Then, as for the five reaction graphs, global and local features of central metrics were accessed, followed by analysis of the cascade number. As *E. coli* metabolic network is the most accurate and comprehensive metabolic model developed up to date [27, 32], we provided in-depth analyses using reaction-centric network of *E. coli*.

**Table 1:** Metabolic networks and their reaction-centric graphs

Strain (model)	Metabolic network (downloaded)			Reaction-centric graphs (converted)		
	Metabolites	Reactions	Genes	Metabolites	Reactions	Arcs
*Escherichia coli* (iJO1366)	1805	2583	1367	2014	1251	9099
*Bacillus subtilis* (iYO844)	990	1250	844	741	748	6489
*Geobacter metallireducens* (iAF987)	1109	1285	987	835	900	8049
*Klebsiella pneumoniae* (iYL1228)	1658	2262	1229	956	1137	8084
*Saccharomyces cerevisiae* (iMM904)	1226	1577	905	1048	881	10,460

### Toy example: topological roles of centrality metrics in a directed network

In graph theory, various kinds of centrality metrics have been developed, and each of them expresses an individual node’s importance in a network by summarizing relations among the nodes from a different perspective. The most frequently used centrality metrics are degree, betweenness centrality, and clustering coefficient, and each of them detects a central node with a different character. Bridging centrality combines two measurements of betweenness centrality and bridging coefficient. Therefore, it detects nodes which act as the bottlenecks of information flow, as well as the bridges (Additional file 1: Figure S1).

**Fig. 1:**
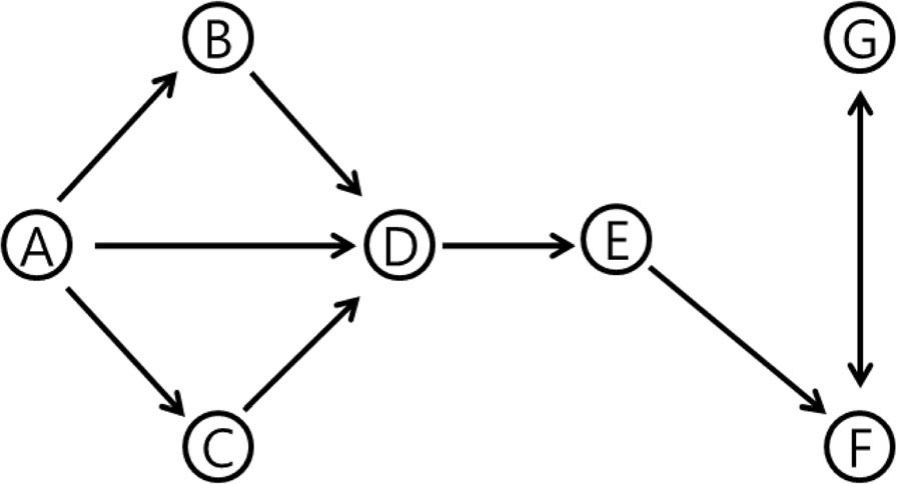
Example of a synthetic network

We explained the properties of the centrality metrics using a synthetic directed network (Fig. 1 and Table 2). Node *A* has the highest cascade number with a cascade set of {B,C,D,E}, meaning that the removal of node *A* closes off the information flow from *A*, to nodes *B*, *C*, *D*, and E. This also implies that the removal of node *A* would result in the separation of local connectivity if the exemplified network belongs to the larger network. A node with high bridging centrality tends to be in the cascade set, for example, node *E* with the highest bridging centrality belongs to the cascade set of node *A*. Nodes *B* and *C* have zero values of betweenness centrality and bridging centrality, as no shortest path passes through them. This implies that a bridging node plays an important role in connecting information flow; it has to be located between modules. The clustering coefficients of nodes *B* and *C* are the highest, as all of their neighbors are still connected after their removal. Node *D* has the highest betweenness centrality as there are many shortest paths passing through it. As node *D* has the highest degree in a module, and is connected to a bridge, it has the lowest bridging coefficient, resulting in a moderate value of bridging centrality. Node *E* has the highest bridging coefficient as it is located between two neighbors with high degrees. It also has high betweenness centrality, resulting in the highest bridging centrality value. This indicates that bridging centrality which was modified for the directed network analysis in this study reflects the importance in considering the topological location of a bridging node well as connection of information flow.

**Table 2:** Centrality values, cascade numbers, and cascade sets shown in Fig. 1

Node	Degree_total_	BC	Br	BrC	CL	C_number	C_set
A	3	0	0.2667	0	0.3333	4	{B,C,D,E}
B	2	0	0.8571	0	0.5	0	∅
C	2	0	0.8571	0	0.5	0	∅
D	4	9	0.1364	1.2273	0.1666	1	{E}
E	2	8	0.8571	6.8571	0	0	∅
F	3	5	0.3333	1.6667	0	1	{G}
G	2	0	1.5000	0	1	0	∅

Each column represents degree in total (Degree_total_), betweenness centrality (BC), bridging coefficient (Br), bridging centrality (BrC), clustering coefficient (CL), cascade number (C_number), and cascade set (C_set)

The toy example demonstrates that both bridging centrality and the cascade number measure a type of influence of a node on the flow of information within a network. Nodes with high bridging centrality are at points where large parts of the graph, called modules, are connected to one another and so have relatively high information flow through them. Nodes with high cascade number will have locally large influence as they have many downstream nodes that depend on them, which means that they have substantial control of information flow in their neighborhood.

### Global topology in the reaction-centric metabolic graphs

There are many ways to translate metabolites and reactions into a graph [33]. In many cases, metabolic networks have been represented as a metabolite-centric graph with metabolites as nodes and reactions as arcs [23–25]. In this study, we represented a metabolic network as a directed reaction-centric graph (reaction graph, hereafter) with reactions as nodes and metabolites as arcs.

To measure modularity in each of the five reaction graphs, we generated 1000 random networks in which the numbers of in-degree and out-degree are set to be those of the corresponding reaction graph. Modularity is widely used to measure how strongly a network is segregated into modules [34], and is defined as the fraction of the arcs that belong within the given modules minus the expected fraction if arcs were distributed at random. All the five reaction graphs were strongly modularized (Additional file 1: Table S1). For example, the modularity in the *E. coli* reaction graph (0.6103) was significantly higher (*P*-value = 0) than those in the degree-matched random networks (mean modularity of 0.2009 and standard deviation of 0.003).

**Fig. 2:**
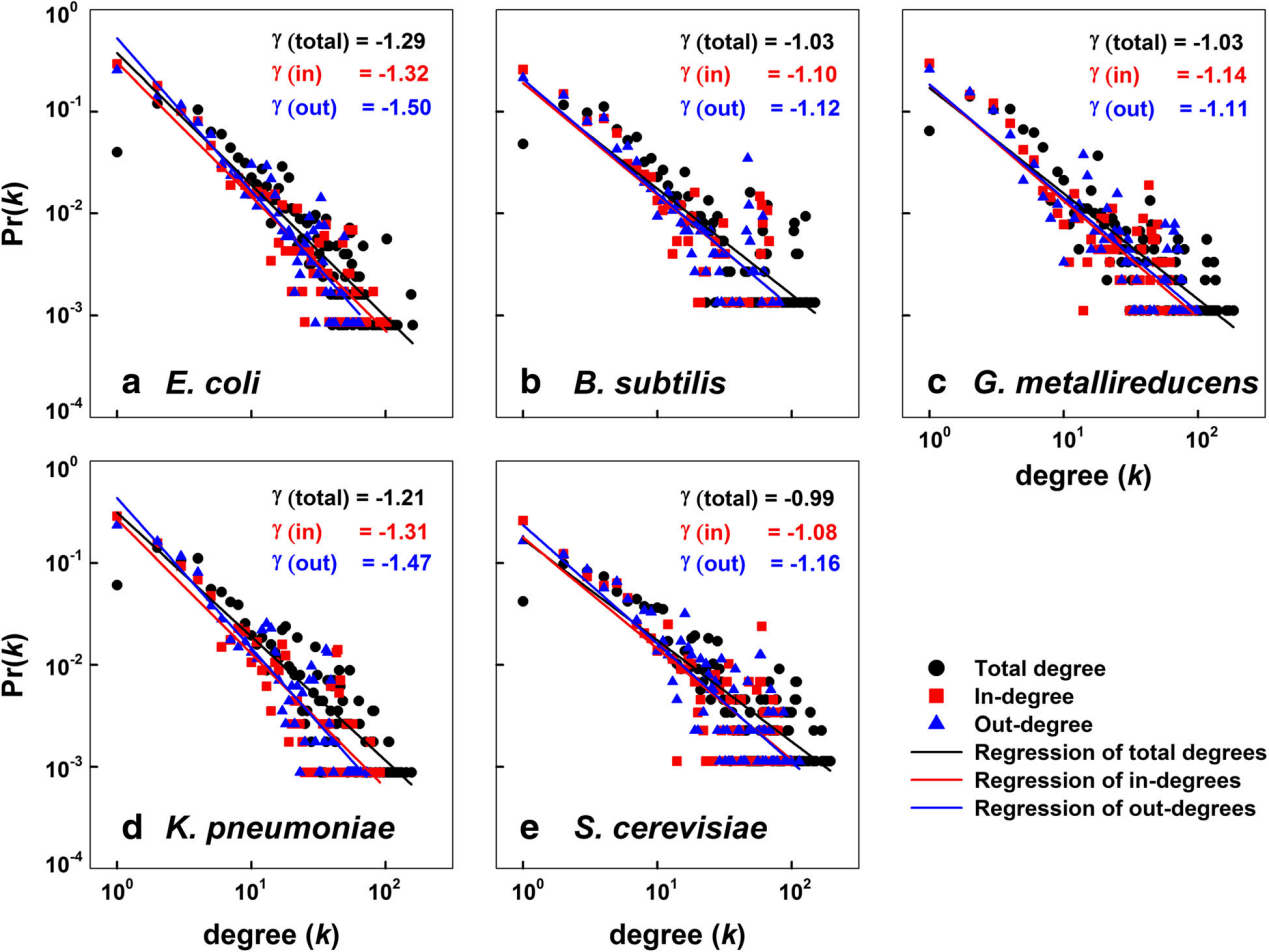
Degree distribution in the reaction-centric metabolic networks. (**a**) *Escherichia coli* (iJO1366), (**b**) *Bacillus subtilis* (iYO844), (**c**) *Geobacter metallireducens* (iAF987), (**d**) *Klebsiella pneumonia* (iYL1228), and (**e**) *Saccharomyces cerevisiae* (iMM904). In-degree (denoted as a red square), out-degree (blue triangle), or total-degree (black circle) was plotted against their probabilities on logarithmic scales

In the five reaction graphs studied, the degree (*k*) distributions of in-, out- and total-degrees followed a power-law (Fig. 2). For example, in the *E. coli* reaction graph, the degree distributions of in-, out- and total-degrees followed a power-law, with γ _in_ = − 1.32, γ _out_ = − 1.50, and γ _total_ = − 1.29, respectively. These indicate that the reaction graph is scale-free, characterized by a small number of heavily connected reaction nodes (hubs).

### Relation of centrality metrics and reaction essentiality

Central metrics can give a ranking of nodes according to their importance in a network. To address biological importance of reactions ranked highly with each central metric, we calculated and compared proportions of the predicted essential reactions in the top 5% of high degree, betweenness, and bridging centralities in the five reaction graphs (Table 3). The essential reactions were predicted using FBA which is a constrained optimization method based on reaction stoichiometry and steady-state assumption [35]. Reactions with high bridging centralities tended to be essential, compared to those with high degree centralities. The exception was the reaction graph of *K. pneumoniae* where the percentages of essential reactions with each centrality metric were almost same.

**Table 3:** Proportions of the predicted essential reactions in the top 5% of reactions with high centralities in the reaction-centric metabolic networks

Centrality	*E. coli* (iJO1366)	*B. subtilis* (iYO844)	*G. metallireducens* (iAF987)	*K. pneumoniae* (iYL1228)	*S. cerevisiae* (iMM904)
Betweenness	37.0%(23/62)	51.3%(19/37)	48.8%(22/45)	28.0%(16/57)	29.5%(13/44)
Bridging	46.7%(29/62)	45.9%(17/37)	71.1%(32/45)	29.8%(17/57)	45.4%(20/44)
Degree	22.5%(14/62)	33.3%(12/36)	16.2%(7/43)	28.5%(16/56)	9.0%(4/44)

Each cell denotes % essential reactions (Number of essential reaction / Number of the top 5% of reactions with high centrality)

**Fig. 3:**
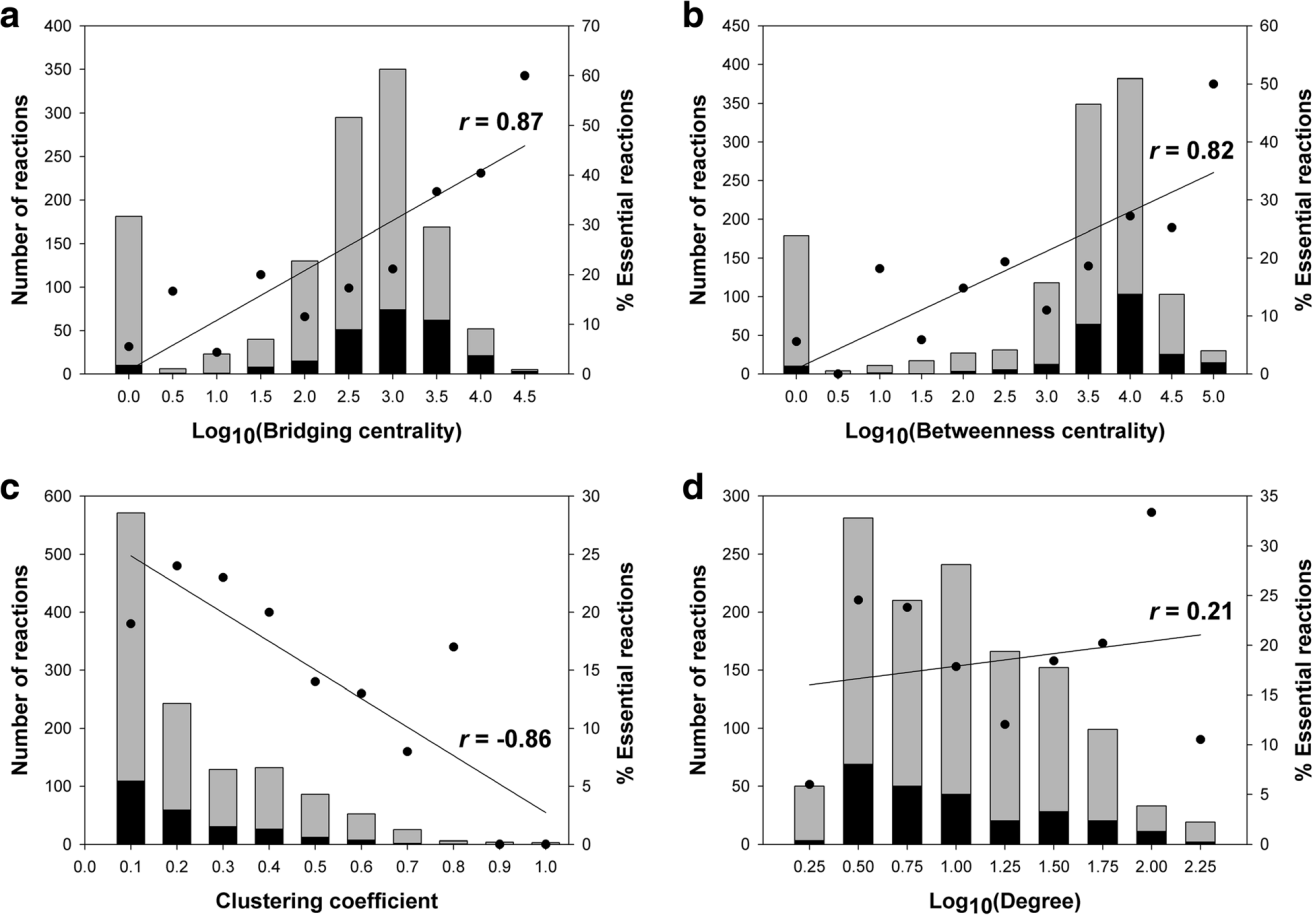
Number distributions of total reactions and essential reactions according to each of the centrality measures in the reaction-centric network of *E. coli*. (**a**) bridging centrality, (**b**) betweenness centrality, (**c**) clustering coefficient, and (**d**) total degree. In each stacked bar, the numbers of predicted essential and non-essential reactions are colored in black and gray, respectively, and their summation is equal to the number of total reactions in *E. coli*. A reaction was considered essential if when its removal from the model led to a growth rate less than the default threshold of 5% of the growth objective value simulated for the wild type strain. The percentage of essential reactions among the total reactions is denoted as a black circle

To expand insights on the influences of each centrality metrics (bridging centrality, betweenness centrality, clustering coefficient, and degrees) on the reaction graph of *E. coli*, numbers of total reactions and essential reactions were plotted according to each of the centrality metrics in the *E. coli* reaction graph (Fig. 3). Reaction deletion simulation by FBA predicted 246 out of the total 1251 reactions to be essential. Among them, 29 were ranked in the top 5% of high bridging centralities (*P*-value = 1.52 × 10^− 7^) and 23 were listed in the top 5% of high betweenness centralities (*P*-value = 2.86 × 10^− 4^). Reactions with high bridging centrality tended to be essential (correlation coefficient (*r*) between bridging centrality and percentage of essential reactions = 0.87) (Fig. 3a). For example (Additional file 1: Figure S2a), among the reactions with high bridging centralities, DHDPRy and HSK were identified as essential reactions by FBA, and were placed on the bridges branched from ASAD to synthesize lysine and threonine, respectively. They also connected each pathway to the reaction which produced input metabolites for the synthesis of the target. Moreover, HSK was located on the tree, which comprised cascade sets leading with ASAD. In case of another example (Additional file 1: Figure S2b), RBFSb and RBFSa were identified as essential reactions by FBA, and they were located on the linear pathway of riboflavin biosynthesis. Interestingly, they were connected with the cascade set that had a leading reaction GTPCI. Reactions with high betweenness centrality tended to be essential as well (*r* = 0.82) (Fig. 3b). The reactions with high clustering coefficients tended to be non-essential (*r* = − 0.86) (Fig. 3c), since in their absence, there was an alternative connection between their neighbors. Unexpectedly, the degree and percentage of essential reactions was not correlated (*r* = 0.21) (Fig. 3d). Reaction deletion simulation showed that the average degree of essential reactions was 14.34, which was quite close to the average degree of all reactions (14.54). This indicates that reactions with high degree tend to have back up pathways or alternative pathways, which acted as substitutes when the high degree reaction was removed.

As illustrated in the synthetic network (Fig. 1 and Table 2), the modified bridging centrality detected nodes functioning as bottlenecks of information flow, as well as the bridges. One of the major differences between nodes having high bridging centrality and high betweenness centrality is their position in the network. For example, in the reaction graph of *E. coli*, while nodes having high betweenness centrality tended to belong to the densely connected modules (such as the pyruvate metabolism pathway or citric acid cycle) (Additional file 1: Table S2), nodes having high bridging centrality were located on bridges between local biosynthesis modules with a few connections (mostly cofactor and prosthetic group biosynthetic pathways) (Additional file 1: Table S3). Moreover, nodes having high bridging centrality have a much lesser metabolic flux value from FBA of wild-type *E. coli* than the nodes having high betweenness centrality. For a node to have high bridging centrality, the node itself has to have a low degree while its neighbors have relatively high degrees. Majority of such cases were found in reactions involved in cofactor biosynthesis. Cofactors are non-protein chemical compounds required for activity of some enzymes. They participate in catalysis, however, are not used as substrates in the enzymatic reactions. In many cases, cofactors are required in minute amounts, and their cellular compositions are very low. For example, serial reactions of RBFSa and RBFSb for riboflavin (vitamin B_2_) biosynthesis showed high bridging centrality scores in the *E. coli* reaction graph. Riboflavin can be synthesized by other six reactions using the reduced form of riboflavin (rbfvrd), which needs to be converted from riboflavin by NAD(P)H-associated reactions. RBFSb is the only riboflavin biosynthetic reaction which does not use rbfvrd. As the riboflavin has stoichiometry of 0.000223 in the *E. coli* growth objective function, the metabolic flux on RBFSb was quite small (0.0004 mmol/gDCW/h) in FBA of the wild-type *E. coli*, although RBFSb was essential predicted by the reaction deletion simulation.

### Analysis of cascade sets and cascade numbers

In evaluating the local influence of a node, it is logical to say that the node had a high degree of control over information flow if its deletion or inactivation deprived its downstream neighbors of information flow within a network. In this study, we developed the cascade algorithm based on counting of nodes which are closed off from the information flow when a particular node is removed. Thus, the cascade number of a node can measure the local controllability for the node. To address the importance of a cascade number in the reaction-centric metabolic networks, we checked whether the removal of a leading reaction node generating a cascade set led to no growth by the reaction deletion simulation of the metabolic network models. Percentage of those essential leading cascade reactions in the total leading cascade reactions were calculated, according to the cascade number (Table 4). In all the five graphs, more than half reactions had zero cascade numbers and didn’t belong to any cascade sets of other reactions. In other words, more than half reactions neither affected network flows when removed. This indicates that majority of reactions did not have any influence over their local connectivity.

**Table 4:** Proportions of essential leading cascade reactions according to the cascade number in the reaction-centric metabolic networks

Reaction graphs from	Cascade number									*r*
	0	1	2	3	4	5	6	> 7	Total	
*E. coli* (iJO1366)	13.4% (94/697)	29.1% (37/127)	30.7% (8/26)	47.6% (10/21)	15.3% (2/13)	25.0% (1/4)	50.0% (1/2)	100% (4/4)	17.5% (157/894)	0.68
*B. subtilis* (iYO844)	22.4% (101/450)	32.2% (19/59)	50.0% (7/14)	83.3% (5/6)	100% (3/3)	ND	50.0% (1/2)	57.1% (4/7)	25.8% (140/541)	0.43
*G. metallireducens* (iAF987)	28.7% (136/473)	65.1% (56/86)	50.0% (13/26)	61.5% (16/26)	54.5% (6/11)	66.6% (2/3)	100% (4/4)	100% (1/1)	37.1% (234/630)	0.86
*K. pneumoniae* (iYL1228)	10.4% (65/620)	28.5% (30/105)	19.3% (6/31)	60.0% (6/10)	41.1% (7/17)	66.6% (2/3)	100% (1/1)	33.3% (2/6)	15.0% (119/793)	0.63
*S. cerevisiae* (iMM904)	10.3% (54/520)	14.4% (11/76)	37.5% (9/24)	41.6% (5/12)	33.3% (2/6)	50.0% (1/2)	50.0% (1/2)	33.3% (1/3)	13.0% (84/645)	0.72

Each cell denotes % essential leading cascade reactions (No. essential leading cascade reactions / No. of total leading cascade reactions). Last column indicates correlation coefficient (*r*) between cascade numbers and % essentialities

Nodes with higher cascade numbers tended to be essential (*r* > 0.63) (Table 4). The exception was the reaction graph converted from iYO844 of *B. subtilis* (*r* = 0.43), mainly due to the presence of non-essential reactions having high cascade numbers. Interestingly, leading cascade reactions became to be essential or not, depending on whether the growth objective function of a metabolic network included the metabolite(s) associated with the cascade set. For example, cascade set reactions by GLUTRS make uroporphyrinogen III (uppg3) which is required to make prosthetic group of siroheme (sheme) (Additional file 1: Figure S2c). Cascade numbers of GLUTRS are 7 and 10 in the reaction graphs of iJO1366 (*E. coli*) and iYO844 (*B. subtilis*), respectively. From the reaction deletion simulation, GLUTRS was essential in iJO1366 and was non-essential in iYO844. The discrepancy in the essentiality of the same reaction in different metabolic models was casused by that sheme was included only in the the growth objective function of iJO1366. In other words, since the growth objective function of iJO1366 contained sheme, growth cannot occur without GLUTRS, and thus, GLUTRS is essential in iJO1366. However, GLUTRS is non-essential in iYO844 whose growth objective function does not have sheme. This example demonstrates that essentiality of a node with a high cascade number can be used in refining a metabolic network model.

When the *E. coli* reaction graph was analyzed using the cascade algorithm, 959 out of 1251 reactions had zero cascade number, implying that most reactions do not have any influence over their local connectivity. Twenty-three reactions had cascade number of ≥4, and each had independent cascade sets forming acyclic subnetworks (Additional file 1: Table S4). Out of the 23 leading cascade reactions, 8 were predicted to be essential by the reaction deletion simulation. Remarkably, all the reactions with a cascade number of 7 (MECDPDH5, ASAD, GTPCI, and GLUTRS) were predicted to be essential, indicating that their removal will result in severe system failure (Table 5). For example (Additional file 1: Figure S2a), the reaction ASAD (catalyzed by aspartate-semialdehyde dehydrogenase) generates ‘aspsa’ (L-aspartate-semialdehyde), which is involved in both the lysine biosynthesis and homoserine biosynthesis. Its cascade set has seven member reactions performing the intermediate steps in the biosynthetic pathway of branched-chain amino acids (leucine, isoleucine, and valine), serine, and glycine. In another example (Additional file 1: Figure S2b), two reactions (GTPCI and GTPCII2) catalyzed by GTP cyclohydrolases, which share the source metabolite GTP, are involved in the first steps of riboflavin biosynthesis and tetrahydrofolate biosynthesis, respectively. The cascade sets of GTPCI, with a cascade number of 7, and GTPCII2, with a cascade number of 3, form subnetworks of tree and linear path, respectively. The cascade set of MECDPDH5 connected the biosynthetic pathways of isoprenoid and ubiquinol. The cascade sets involved many reactions with high bridging centralities, while they had much lesser intersections with reactions with high betweenness centralities (Additional file 1: Figure S3). This is not surprising, considering bridging centrality tended to be placed on bridges between modules with a few connections.

**Table 5:** Cascade sets with the highest cascade number in the reaction-centric metabolic network of *E. coli*

Leading cascade reaction (Cascade number)	Cascade set	Subsystem (function)	Subnetwork type^a^	Flux^b^	Essentiality^c^
MECDPDH5 (7)	DMPPS, IPDPS, OCTDPS, UDCPDPS, DMATT, IPDDI, GRTT	Cofactor and prosthetic group biosynthesis (Connecting Isoprenoid and ubiquinol)	Other	0.002	T
ASAD (7)	THRAi, THRD, THRD_L, HSDy, THRS, HSK, THRTRS	Threonine and lysine metabolism (Junction of lysine and threonine branches)	Tree	−1.050	T
GTPCI (7)	CPH4S, CDGS, DHPTPE, CCGS, CDGR, DNMPPA, DNTPPA	Cofactor and prosthetic group biosynthesis (Folate synthesis and producing ‘glycit’)	Tree	0.002	T
GLUTRS (7)	GLUTRR, G1SAT, PPBNGS, HMBS, UPP3S, UPPDC1, CPPPGO	Cofactor and prosthetic group biosynthesis (Importing glu-L to synthesize hemeO biosynthesis)	Linear path	0.004	T

Abbreviations can be found in BiGG database (http://bigg.ucsd.edu/)

^a^Drawn for the leading cascade reaction and its cascade set reactions; All the subnetwork are acyclic subnetworks classified into three types: tree, linear path, and other (neither linear path nor tree)

^b^Metabolic flux value from FBA of wild-type *E. coli* (mmol/gDCW/h)

^c^Essentiality of a reaction predicted from the reaction deletion simulation

The idea of breakage of information flow was also implemented in topological flux balance (TFB) failure algorithm based on flux balance criterion which was devised to search bidirectional failure along the directed bipartite metabolic graph having two types of nodes (metabolites and reactions) [36]. Under the steady-state assumption of a metabolic network, TFB detects large-scale cascading failure where the removal of a single reaction can delete downstream neighbored nodes which lose all the inputs as well as upstream neighbors which lose all the outputs [36], and thus, it is more suitable for measuring global robustness of a directed bipartite network. By contrast, the cascade algorithm developed in this study searches only the downstream neighbors which lose all the inputs when a specific node is removed, focusing on the local cascading failure in a directed network.

## Discussion

Topological analysis of a metabolic network provides valuable insights into the internal organization of the network and topological roles of individual nodes [1, 9]. Detection of central nodes in asymmetrically directed biological networks depends on biological questions about the global and local topology of the network. Various centrality metrics seek to quantify an individual node’s prominence in a network by summarizing structural relations among the nodes, although most centrality metrics correlate with degree indicating that highly connections among nodes are important. In this study, for the topological analysis of metabolic networks, we applied various centrality metrics to directed reaction-centric graphs of the five phylogenetically distant organisms. Degree centrality, betweenness centrality, clustering coefficient, and modularity were found to be useful in discovering global topological properties and modular structures of the reaction graphs. To explain connections between modules and local connectivity in directed reaction-centric graphs, we modified the bridging centrality and developed the cascade number. We demonstrated that the cascade algorithm and the modified bridging centrality can identify cascade subnetworks controlling local information flow and irreplaceable bridging nodes between functional modules, respectively.

When metabolic and biochemical networks are represented as metabolite graphs, they have been known to be scale-free and small-world [3, 24, 37]. In this work, we found that the distribution of the degree of the reaction graphs of all the five phylogenetically distant microorganisms followed a power law (Fig. 2). This agrees with previous report that reaction graphs of cancer metabolic networks followed power law degree distribution [13]. However, this is in contrast with a previous work showing that the *E. coli* reaction graph with undirected edges was not scale-free [38]. This discrepancy can be attributed to the differences in network size and directionality: we used a directed reaction graph of *E. coli* metabolic network that is much bigger than that in the previous study [38], and considered the directionality of the reaction flow, which added more nodes and information to the network.

In this study, we found that reaction nodes linking between modules needed not be hubs with high degree. This is contrasting to the metabolite hubs which connect modules in metabolite-centric metabolic networks [3, 24]. There were two types of connections among the modules in the reaction graphs: the bottleneck with high betweenness centrality and the bridge with high bridging centrality. The high betweenness reactions had the potential to disconnect the network and damage the organism’s growth rate when removed. Although betweenness centrality was not correlated with degree, the degrees of high betweenness reactions were relatively high or medium (Additional file 1: Table S2), suggesting that betweenness centrality would measure global connectivity among central modules with many connections. On the other hand, bridging centrality could detect nodes which were placed on the bridges between local biosynthesis modules with a few connections (Additional file 1: Table S3).

We developed a novel metric, called the *cascade number*, to identify local connectivity structures in directed graphs. The cascade number can count how many reactions shut down if one reaction is perturbed at a steady state, and can measure their influence over local connectivity for metabolite flow. Perturbation of a node with a high cascade number could alter the local route of metabolic process, or cause damage to the metabolic system. In the *E. coli* reaction graph, 959 out of the 1251 total reactions had the cascade number of zero, which implies that most reactions did not have any influence over their local connectivity. It has been known that universal metabolic pathways across species, such as citric acid cycle and glycolytic pathways, have relatively few essential reactions [39, 40]. This fact indicates that important reactions are more likely to have a backup pathway [40, 41], and therefore, the cascade number of such reactions tended to be low or zero. By contrast, nodes with higher cascade numbers tended to be essential, implying that their removal will result in severe breakage of information flow in a metabolic network (Table 4 and Additional file 1: Table S4).

Both bridging centrality and the cascade number are local properties, reflecting local information flow within a metabolic network. Bridging centrality can be used to locate nodes in the network that lie on the boundaries of modules within a network. The nodes with high bridging centrality, even though they are located with local information, can have global importance, forming breakpoints in the information flow. The importance of the cascade number is also potentially global, though less so than bridging centrality. A node with a high cascade number is a node with larger degree of influence on the network. The global impact of a node with high local influence can be accessed by simulation or biological experimentation. Knowing the nodes with a large cascade number informs the design of such experiments: these nodes are more likely than others to have a large influence and can be looked at first.

## Conclusions

In this study, we explored topological features of individual reaction nodes in reaction-centric metabolic networks from global and local perspectives. In particular, we demonstrated that the cascade number and the modified bridging centrality can identify reaction nodes that control the local information flow in the reaction graphs. Identification of central connectors between local modules with the modified bridging centrality, together with local flow connectivity, which was ascertained with the cascade algorithm, is critical to understand how metabolic pathways are assembled. A metabolic network is a map that assembles central and local biosynthesis pathways where the metabolites run through the reactions. Identifying reaction nodes and their associated genes important in global and local connectivity between modules can be useful to prioritize targets in the fields of metabolic engineering and medicine.

## Methods

### Centrality metrics in a directed network

Several centrality metrics have been developed to identify important components in a network from different centrality viewpoints [1]. Among them, we applied the clustering coefficient and betweenness centrality to the analysis of directed networks. As bridging centrality had been developed for undirected networks [11], we modified it to be applied for directed networks.

### Clustering coefficient

The neighbors of a node *i* are defined as a set of nodes connected directly to the node *i*. The clustering coefficient of a node in a network quantifies how well its neighbors are connected to each other [42]. The clustering coefficient of a node *i*, *C(i)*, is the ratio of the number of arcs between the neighbors of *i* to the total possible number of arcs between its neighbors. For a directed network, *C(i)* can be calculated as:

$$ C(i)=\frac{n_i}{k_i\left({k}_i-1\right)}, $$

where *n*_*i*_ is the number of arcs between neighbors of the node *i*, and *k*_i_ is the number of neighbors of the node *i*. The closer the clustering coefficient of a node is to 1, the more likely it is for the node and its neighbors to form a cluster. By definition, it measures the tendency of a network to be divided into clusters, and thus, is related to network modularity. The majority of biological networks have a considerably higher average value for the clustering coefficient in comparison to random networks, indicating that they have a modular nature [1].

### Betweenness centrality

The betweenness centrality of a node is the fraction of shortest paths from all nodes to all others that pass through the particular node [10]. The betweenness centrality of a node *i*, *B*(*i*)*,* is calculated as:

$$ B(i)=\sum \limits_{j\ne i\ne k}\frac{\sigma_{jk}(i)}{\sigma_{jk}}, $$

where *σ*_*jk*_ is the total number of shortest paths from node *j* to node *k*, and *σ*_*jk*_(*i*) is the total number of those paths that pass through node *i*. The higher the betweenness centrality of a node is, the higher the number of shortest paths that pass through the node. A node with a high betweenness centrality has a large influence on the information flow through the network, under the assumption that reaction flow follows the shortest paths [43]. The node with a high betweenness centrality tends to be a linker between modules, and has often been called a *bottleneck* in the network [44]. Although a bottleneck node does not necessarily have many interactions like a hub node, its removal often results in a higher fragmentation of a network, than when a hub node is removed.

### Modification of bridging centrality

The bridging centrality identifies bridging nodes lying between densely connected regions called modules [11]. The bridging centrality of node *i*, *BrC*(*i*), is calculated as the product of the betweenness centrality, *B*(*i*), and the bridging coefficient, *BC*(*i*), which measure the global and local features of a node, respectively [11].

$$ BrC(i)=B(i)\times BC(i) $$

Previously, the bridging coefficient in an undirected network was defined [11] as:

$$ BC(i)=\frac{{\left( degree(i)\right)}^{-1}}{\sum_{j\  in\ \varLambda (i)}{\left( degree(j)\right)}^{-1}}, $$

where *Λ*(*i*) is the set neighbors of the node *i*.

In a directed network where the information flows through a node, the node needs to have both incoming and outgoing edges. Thus, we modified the bridging coefficient in a directed network as:

$$ BC(i)=\left\{\begin{array}{c}\ \frac{{\left( degre{e}_{total}(i)\right)}^{-1}}{\sum_{j\  in\ \varLambda (i)}{\left( degre{e}_{total}(j)\right)}^{-1}}\kern0.5em if\ degre{e}_{in}(i)\ne 0\  and\ degre{e}_{out}(i)\ne 0\\ {}0\kern9.5em otherwise\end{array}\right., $$

where *degree*_*total*_(*i*) is the sum of *degree*_*in*_(*i*) and *degree*_*out*_(*i*) of node *i*.

By definition, for a node to have a high bridging coefficient, degrees of the node and the number of its neighbors have to be low and high, respectively. Both betweenness centrality and bridging coefficient have a positive effect on bridging centrality. These indicate that from the perspective of information flow, a good example of a node with high bridging centrality would be a bridge in the form of a path with length two, uniquely delivering information between neighbors that themselves have high degrees (Additional file 1: Figure S1).

### Development of a cascade algorithm

We devised a cascade algorithm for detecting how many nodes are closed off from information flow when a particular node is removed in a directed network. If a node is locked down or suffers an accidental shutdown, such a change is propagated through the network. Any nodes dependent on the failed node cannot receive the information if there are no alternate path(s) bypassing the failed node. We defined the “*cascade set*” of a node as the set of nodes that cease to receive information when the node fails, and the “*cascade number*” of a node as the number of nodes in the cascade set. For two cascade sets *A* and *B*, if a leading cascade node generating *A* belongs to *B*, *A* is included in *B*. A cascade set becomes *independent* if its member nodes are not included in any other cascade sets. A node generating an independent cascade set was referred to as a “*leading cascade node*”.

Let a directional network be an ordered pair, (*V*, *A*), where *V* is the set of nodes and *A* is the set of arcs of the network. Then, the cascade set and cascade number are computed by the following algorithm:

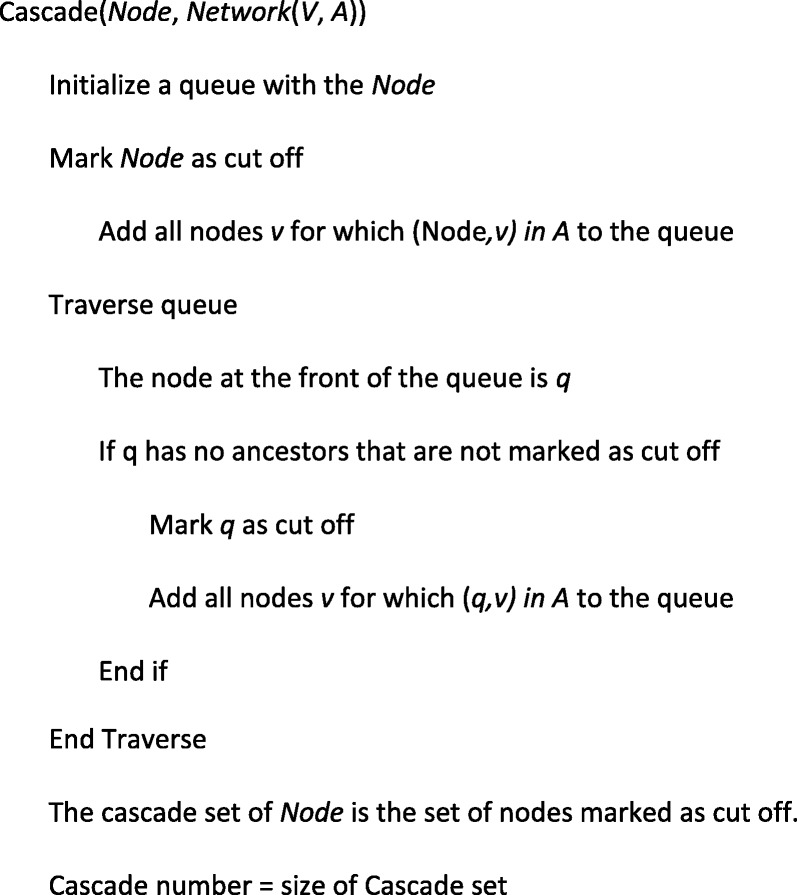



### Graph representation of a directed reaction-centric metabolic network

The reaction graph was represented as a directed graph with metabolic reactions as nodes and metabolites as arcs. The reactions and metabolites were collected from the metabolic network models of *E. coli* (iJO1366) [27], *B. subtilis* (iYO844) [28], *G. metallireducens* (iAF987) [29], *K. pneumonia* (iYL1228) [30], and *S. cerevisiae* (iMM904) [31] (Table 1), which were downloaded from the BIGG database [45] in the SBML file format. For each of the metabolic network models, the collected reactions and metabolites were used to reconstruct the reaction graph (Table 1). For example, 1805 unique metabolites and 2583 metabolic reactions in iJO1366 of *E. coli* were reconstructed to the reaction graph consisting of 1251 nodes (reactions) and 9099 arcs associated with 2014 metabolites. Adjacency matrices of the five reaction graphs converted from the downloaded metabolic network models are provided as Additional file 2.

A reaction graph is *G* = (*V*, *A*) where *V* is a set of reaction nodes, and *A* is a set of *V*’s arcs. There exists an arc from the reaction B to the reaction C when a product of B is consumed by C. For example, consider following three consecutive reactions:

ASAD: 4pasp ↔ aspsa

HSDy: aspsa ↔ hom-L

HSK: hom-L → phom

The corresponding arcs are ASAD→HSDy, HSDy→ASAD, and HSDy→HSK (i.e., ASAD↔HSDy→ HSK), where two consecutive reversible reactions of ASAD and HSDy form the directed cycle with length of two.

Currency metabolites such as ATP, NAD, and H_2_O are ubiquitously associated with metabolic reactions. However, they are not incorporated into the final products. As pathways routing through the currency metabolites result in a biologically meaningless short path length, the currency metabolites were removed [24, 38, 46]. Similarly, transport and exchange reactions occurring at the cell boundary were removed, as they do not affect any relationship or reaction flow among intracellular reactions, while they inflate the size of the network and the average path length, and weaken the modular structure of intracellular connectivity.

In the converted reaction graph, the degree of a reaction node is the number of other reactions that produce (or consume) metabolites which are consumed (or produced) by the reaction node. For example, consider a reaction AACPS1 (ACP[c] + atp[c] + ttdca[c] - > amp[c] + myrsACP[c] + ppi[c]). AACPS1 has two metabolites of ACP[c] and ttdca[c] as reactants, and one metabolite of myrsACP[c] as a product. (Recall that the currency metabolites of atp[c], amp[c], and ppi[c] were removed in the reaction graph.) ACP[c] and ttdca[c] are produced from other 57 reactions, and myrsACP[c] is consumed in 7 reactions. Therefore, the in-degree and out-degree of the reaction node AACPS1 are 57 and 7, respectively.

### Simulation of reaction essentiality in the metabolic networks

To identify reactions which are essential for cell growth, flux balance analysis (FBA) [47] was performed to simulate cell growth when each reaction was removed from each metabolic network model. The default flux boundaries in the downloaded SBML files were used for the simulation condition and maximum growth rate was for the objective function. In FBA, the allowed nutrients for iJO1366 (*E. coli*) were Ca^2+^, Cl^−^, CO_2_, Co^2+^, Cob(I)alamin, Cu^2+^, Fe^2+^, Fe^3+^, glucose, H^+^, H_2_O, HPO_4_^2−^, K^+^, Mg^2+^, Mn^2+^, MoO_4_^2−^, Na^+^, NH_4_^+^, Ni^2+^, O_2_, selenate, selenite, SO_4_^2−^, tungstate, and Zn^2+^; for iYO844 (*B. subtilis*), Ca^2+^, CO_2_, Fe^3+^, glucose, H^+^, H_2_O, HPO_4_^2−^,K^+^, Mg^2+^, Na^+^, NH_4_^+^, O_2_, and SO_4_^2−^; for iYL1228 (*K. pneumoniae*), Ca^2+^, Cl^−^, CO_2_, Co^2+^, Cu^2+^, Fe^2+^, Fe^3+^, glucose, H^+^, H_2_O, HPO_4_^2−^, K^+^, Mg^2+^, Mn^2+^, MoO_4_^2−^, Na^+^, NH_4_^+^, O_2_, SO_4_^2−^, tungstate, and Zn^2+^; for iMM904 (*S. cerevisiae*), Fe^2+^, glucose, H^+^, H_2_O, HPO_4_^2−^, K^+^, O_2_, Na^+^, NH_4_^+^, and SO_4_^2−^; and for iAF987 (*G. metallireducens*), acetate, Cd^2+^, Ca^2+^, Cl^−^, chromate, CO_2_, Co^2+^, Cu^+^, Cu^2+^, Fe^2+^, Fe^3+^, H^+^, H_2_O, HPO_4_^2−^, K^+^, Mg^2+^, Mn^2+^, MoO_4_^2−^, Na^+^, N_2_, NH_4_^+^, Ni^2+^, SO_4_^2−^, SO_3_^2−^, tungstate, and Zn^2+^. A reaction was considered essential if when its removal from the model led to a growth rate less than the default threshold of 5% of the growth objective value simulated for the wild type strain [48]. The simulation was carried out using COBRA toolbox version 2.0 [49] in MATLAB R2016a (Mathworks Inc.).

## Additional files

Additional file 1: **Table S1.** Modularity and scale-freeness of the reaction-centric metabolic networks; **Table S2.** The top 2% of reactions with high betweenness centrality in the reaction-centric metabolic network of *E. coli*; **Table S3.** The top 2% of reactions with high bridging centrality scores in the reaction-centric metabolic network of *E. coli*; **Table S4.** Cascade sets (with a cascade number of ≥4) and their characteristics in the reaction-centric metabolic network of *E. coli*; **Figure S1.** Example of a bridge node (*n*) with high bridging centrality; **Figure S2.** Examples of cascade sets consisting of a linear path and a tree; **Figure S3.** Comparison of reactions with high centralities identified in the reaction-centric metabolic network of *E. coli*. (DOCX 221 kb)

Additional file 2: Adjacency matrices of the five reaction graphs. (XLSX 13419 kb)

## Abbreviation

FBA: Flux balance analysis
