# Supplementary material for: Identification of critical connectors in the directed reaction-centric graphs of microbial metabolic networks
Source: BMC Bioinformatics. 2019 Jun 13;20:328. doi: 10.1186/s12859-019-2897-z (PMC6567475; doi:10.1186/s12859-019-2897-z)
Supplement: Supplementary file 1 — Table S1. Modularity and scale-freeness of the reaction-centric metabolic networks; Table S2. The top 2% of reactions with high betweenness centrality in the reaction-centric metabolic network of E. coli; Table S3. The top 2% of reactions with high bridging centrality scores in the reaction-centric metabolic network of E. coli; Table S4. Cascade sets (with a cascade number of ≥4) and their characteristics in the reaction-centric metabolic network of E. coli; Figure S1. Example of a bridge node (n) with high bridging centrality; Figure S2. Examples of cascade sets consisting of a linear path and a tree; Figure S3. Comparison of reactions with high centralities identified in the reaction-centric metabolic network of E. coli. (DOCX 221 kb) [file 12859_2019_2897_MOESM1_ESM.docx]

**Additional file 1**

**Identification of critical connectors in the directed reaction-centric graphs of microbial metabolic networks**

Eun-Youn Kim, Daniel Ashlock and Sung Ho Yoon

**Table S1. Modularity and scale-freeness of the reaction-centric metabolic networks**

| **Strain** | **Modularity (*P*-value)** | **Degree distributions** (**Pr(*k*) ≈ *k*^-γ^ )** | | |
| --- | --- | --- | --- | --- |
|  |  | **Total degree (γ*_tota_*_l_)** | **In- degree**  **(γ _in_** **)** | **Out-degree**  **(γ*_out_*)** |
| ***E. coli* (iJO1366)** | 0.6103 (0) | -1.29 | -1.32 | -1.50 |
| ***B. subtilis* (iYO844)** | 0.5622 (0) | -1.03 | -1.10 | -1.12 |
| ***G. metallireducens* (iAF987)** | 0.5179 (0) | -1.03 | -1.14 | -1.11 |
| ***K. pneumoniae* (iYL1228)** | 0.6266 (0) | -1.21 | -1.31 | -1.47 |
| ***S. cerevisiae* (iMM904)** | 0.5705 (0) | -0.99 | -1.08 | -1.16 |

**Table S2. The top 2% of reactions with high betweenness centrality in the reaction-centric metabolic network of *E. coli***

| **BC rank^a^** | **Reaction** | **Subsystem** | **Flux^b^** | **in/out-degree** | **Essentiality^c^** |
| --- | --- | --- | --- | --- | --- |
| 1 | POR5 | Pyruvate metabolism | 0.107 | 91/64 | F |
| 2 | MCOATA | Membrane lipid metabolism | 0.076 | 97/58 | T |
| 3 | ALATA_L | Alanine and aspartate metabolism | -1000 | 72/50 | F |
| 4 | ACOATA | Membrane lipid metabolism | 0 | 102/57 | F |
| 5 | DDPGALA | Alternate carbon metabolism | 0 | 46/22 | F |
| 6 | ASPTA | Alanine and aspartate metabolism | -2.876 | 45/51 | T |
| 7 | TKT1 | Pentose phosphate pathway | 1.167 | 23/17 | F |
| 8 | SERAT | Cysteine metabolism | 0.244 | 57/62 | T |
| 9 | CMPN | Nucleotide salvage pathway | 0 | 29/6 | F |
| 10 | ACGS | Arginine and proline metabolism | 0.291 | 52/33 | T |
| 11 | G3PD2 | Alternate carbon metabolism | -0.137 | 23/56 | T |
| 12 | GLCATr | Alternate carbon metabolism | 0 | 66/48 | F |
| 13 | NDPK2 | Nucleotide salvage pathway | 0.418 | 13/8 | T |
| 14 | GHMT2r | Glycine and serine metabolism | 1.096 | 23/28 | F |
| 15 | PRPPS | Histidine metabolism | -999 | 10/15 | F |
| 16 | UAGAAT | Lipopolysaccharide biosynthesis / recycling | 0.038 | 54/19 | T |
| 17 | GLNS | Glutamate metabolism | 1.779 | 31/23 | T |
| 18 | ADSS | Purine and pyrimidine biosynthesis | 0.294 | 11/8 | T |
| 19 | SUCOA | Citric acid cycle | -1000 | 63/38 | F |
| 20 | CTPS2 | Purine and pyrimidine biosynthesis | 0.158 | 2/39 | T |
| 21 | GLYAT | Glycine and serine metabolism | 0 | 60/113 | F |
| 22 | NDPK1 | Nucleotide salvage pathway | -999 | 11/26 | F |
| 23 | ADK3 | Nucleotide salvage pathway | -1000 | 11/26 | T |
| 24 | AHCYSNS | Methionine metabolism | 0.0004 | 12/5 | F |
| 25 | MALS | Anaplerotic reactions | 0.0007 | 27/39 | F |

^a^Rank of betweenness centrality scores

^b^Metabolic flux value from FBA of wild-type *E. coli* (mmol/gDCW/h)

^c^Essentiality of a reaction predicted from the reaction deletion simulation

Abbreviations can be found in BiGG database (<http://bigg.ucsd.edu/>)

**Table S3. The top 2% of reactions with high bridging centrality scores in the reaction-centric metabolic network of *E. coli***

| **BrC rank^a^** | **Reaction** | **Subsystem** | **Flux^b^** | **in/out-**  **degree** | **Essentiality^c^** |
| --- | --- | --- | --- | --- | --- |
| 1 | DHNCOAS | Cofactor and prosthetic group biosynthesis | 0 | 1/1 | F |
| 2 | RBFSb | Cofactor and prosthetic group biosynthesis | 0.0004 | 1/4 | T |
| 3 | RZ5PP | Cofactor and prosthetic group biosynthesis | -2.084 | 1/1 | F |
| 4 | DHDPRy | Threonine and lysine metabolism | 0.364 | 1/1 | T |
| 5 | MPTSS | Cofactor and prosthetic group biosynthesis | 0.0005 | 1/1 | T |
| 6 | SGSAD | Arginine and proline metabolism | 0 | 1/1 | F |
| 7 | RBFSa | Cofactor and prosthetic group biosynthesis | 0.0009 | 3/1 | T |
| 8 | HEPK2 | Lipopolysaccharide biosynthesis / recycling | -2.33E-29 | 1/1 | F |
| 9 | MALCOAMT | Cofactor and prosthetic group biosynthesis | 1.96E-06 | 4/2 | T |
| 10 | 3OAR100 | Cell envelope biosynthesis | -4.75E-17 | 1/1 | F |
| 11 | HSK | Threonine and lysine metabolism | 0.535 | 1/1 | T |
| 12 | EDD | Pentose phosphate pathway | 0 | 2/1 | F |
| 13 | PPM2 | Alternate carbon metabolism | 0 | 5/6 | F |
| 14 | ALAGLUE | Murein recycling | 0 | 1/1 | F |
| 15 | ASAD | Threonine and lysine metabolism | -1.050 | 2/3 | T |
| 16 | PPCDC | Cofactor and prosthetic group biosynthesis | 0.0006 | 1/1 | T |
| 17 | ADOCBIK | Cofactor and prosthetic group biosynthesis | 0 | 1/1 | F |
| 18 | ALLTN | Nitrogen metabolism | 0 | 1/1 | F |
| 19 | GLUTRR | Cofactor and prosthetic group biosynthesis | 0.004 | 1/2 | T |
| 20 | KARA1 | Valine, leucine, and isoleucine metabolism | -0.859 | 1/1 | T |
| 21 | MOADSUx | Cofactor and prosthetic group biosynthesis | 0.0005 | 2/2 | T |
| 22 | G1SAT | Cofactor and prosthetic group biosynthesis | 0.004 | 1/1 | T |
| 23 | SHKK | Tyrosine, tryptophan, and phenylalanine metabolism | 0.374 | 1/1 | T |
| 24 | HMBS | Cofactor and prosthetic group biosynthesis | 0.0004 | 1/1 | T |
| 25 | 3OAR120 | Cell envelope biosynthesis | -4.75E-17 | 1/1 | F |

^a^Rank of bridging centrality scores

^b^Metabolic flux value from FBA of wild-type *E. coli* (mmol/gDCW/h)

^c^Essentiality of a reaction predicted from the reaction deletion simulation

Abbreviations can be found in BiGG database (<http://bigg.ucsd.edu/>)

**Table S4. Cascade sets (with a cascade number of ≥ 4) and their characteristics in the reaction-centric metabolic network of *E. coli***

| **Leading cascade reaction (Cascade number)** | **Cascade set** | **Subsystem** | **Subnetwork type^a^** | **Flux^b^** | **Essentiality^c^** |
| --- | --- | --- | --- | --- | --- |
| MECDPDH5(7) | DMPPS, IPDPS, OCTDPS, UDCPDPS, DMATT, IPDDI, GRTT | Cofactor and prosthetic group biosynthesis | None | 0.002 | T |
| ASAD(7) | THRAi, THRD, THRD_L, HSDy, THRS, HSK, THRTRS | Threonine and lysine metabolism | Tree | -1.050 | T |
| GTPCI(7) | CPH4S'3, CDGS'2, DHPTPE, CCGS'1, CDGR, DNMPPA, DNTPPA | Cofactor and prosthetic group biosynthesis | Tree | 0.002 | T |
| GLUTRS(7) | CPPPGO, GLUTRR, MAN1PT2, UPP3S, G1SAT, HMBS, PPBNGS | Cofactor and prosthetic group biosynthesis | Linear path | 0.004 | T |
| MPTS(6) | MOCOS, BMOCOS, BWCOS, MPTAT, WCOS, MPTSS | Cofactor and prosthetic group biosynthesis | Tree | 0.0002 | T |
| XAND(6) | URIC, UGLYCH, ALLTAMH, OXAMTC, ALLTN, URDGLYCD | Nucleotide salvage pathway | Tree | 0 | F |
| DDPA(5) | SHKK, DHQS, SHK3Dr, DHQTi, QUINDH | Tyrosine, tryptophan, and phenylalanine metabolism | Tree | 0.374 | T |
| MAN1PT2(5) | GDPMNH, GDPMNP, GMAND, GDMANE, GOFUCR | Cell envelope biosynthesis | Tree | 0 | F |
| 3OAS100(5) | 3OAR100, EAR100x, 'EAR100y, T2DECAI,3HAD100 | Cell envelope biosynthesis | Linear path | 0 | F |
| PYROX(5) | URACPAH, CBMD, POAACR, 3AMACHYD, MSAR | Nucleotide salvage pathway | Tree | 0 | F |
| DKGLCNR1(4) | 2DGULRGx, 2DGULRGy, 2DGULRx, 2DGULRy | Alternate carbon metabolism | Tree | 0 | F |
| OPMEACPS(4) | OPMEACPS, PMEACPE, OPMEACPR, OPMEACPD, EPMEACPR | Cofactor and prosthetic group biosynthesis | Linear path | 1.96E-06 | T |
| PPNCL2(4) | PPCDC, PTPATi, DPCOAK, ACPS1 | Cofactor and prosthetic group biosynthesis | Tree | 0.0005 | T |
| S7PI(4) | GMHEPK, GMHEPPA, GMHEPAT, AGMHE | Lipopolysaccharide biosynthesis / recycling | Linear path | 0 | F |
| 3OAS120(4) | 3OAR120, 3HAD120, EAR120x, EAR120y | Cell envelope biosynthesis | Tree | 0 | F |
| 3OAS121(4) | 3OAR121, 3HAD121, EAR121x, EAR121y | Cell envelope biosynthesis | Tree | 0 | F |
| 3OAS141(4) | 3OAR141, 3HAD141, , EAR141x, EAR141y | Cell envelope biosynthesis | Tree | 0 | F |
| 3OAS160(4) | 3OAR160, 3HAD160, EAR160x, EAR160y | Cell envelope biosynthesis | Tree | 0 | F |
| 3OAS161(4) | 3OAR161, 3HAD161, EAR161x, EAR161y | Cell envelope biosynthesis | Tree | 0 | F |
| 3OAS180(4) | 3OAR180, 3HAD180, EAR180x, EAR180y | Cell envelope biosynthesis | Tree | 0 | F |
| 3OAS181(4) | 3OAR181, 3HAD181, EAR181x, EAR181y | Cell envelope biosynthesis | Tree | 0 | F |
| 3OAS60(4) | 3OAR60, 3HAD60, EAR60x, EAR60y | Cell envelope biosynthesis | Tree | 0 | F |
| 3OAS80(4) | 3OAR80, 3HAD80, EAR80x, EAR80y | Cell envelope biosynthesis | Tree | 0 | F |
| GTPCI2(3)^d^ | DHPPDA2, APRAUR, PMDPHT | Cofactor and prosthetic group biosynthesis | Linear path | 0.0007 | T |

**^a^**Drawn for the leading cascade reaction and its cascade set reactions; All the subnetwork are acyclic subnetworks classified into three types: tree, linear path, and other (neither linear path nor tree)

^b^Metabolic flux value from FBA of wild-type *E. coli* (mmol/gDCW/h)

^c^Essentiality of a reaction predicted from the reaction deletion simulation

^d^This set with a cascade number of 3 was included to be explained in the main text

Abbreviations can be found in BiGG database (<http://bigg.ucsd.edu/>)

**Figure S1. Example of a bridge node (*n*) with high bridging centrality.**


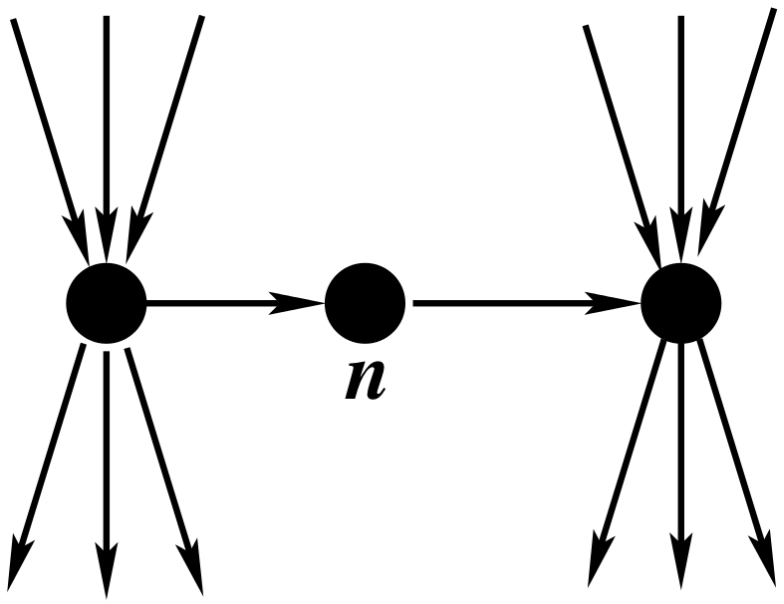


**Figure S2. Examples of cascade sets consisting of a linear path and a tree.** (**a**) ASAD (catalyzed by aspartate-semialdehyde dehydrogenase) with cascade number of 7. Its cascade set is membered in 7 reactions performing intermediate steps in the biosynthetic pathway of branched-chain amino acids (leucine, isoleucine, and valine), serine, and glycine. (**b**) Two GTP cyclohydrolases, GTPCI (GTP cyclohydrolase I) with a cascade number of 7, and GTPCI2 (GTP cyclohydrolase II) with a cascade number of 3, are the first steps in riboflavin biosynthesis and tetrahydrofolate biosynthesis, respectively. (**c**) GLUTRS (glutamyl-tRNA synthetase) with a cascade number of 7 and its cascade set are required to produce upp3 which is required to make sheme. The abbreviation can be found in BiGG database (<http://bigg.ucsd.edu/>). Reactions of the leading cascade reaction and its cascade set are colored in red.

**a**

**b**

**c**

**Figure S3. Comparison of reactions with high centralities identified in  the reaction-centric metabolic network of *E. coli*.** Numbers are for reactions with the top 10% in betweenness and bridging centrality scores, and for reactions with non-zero cascade numbers.

**
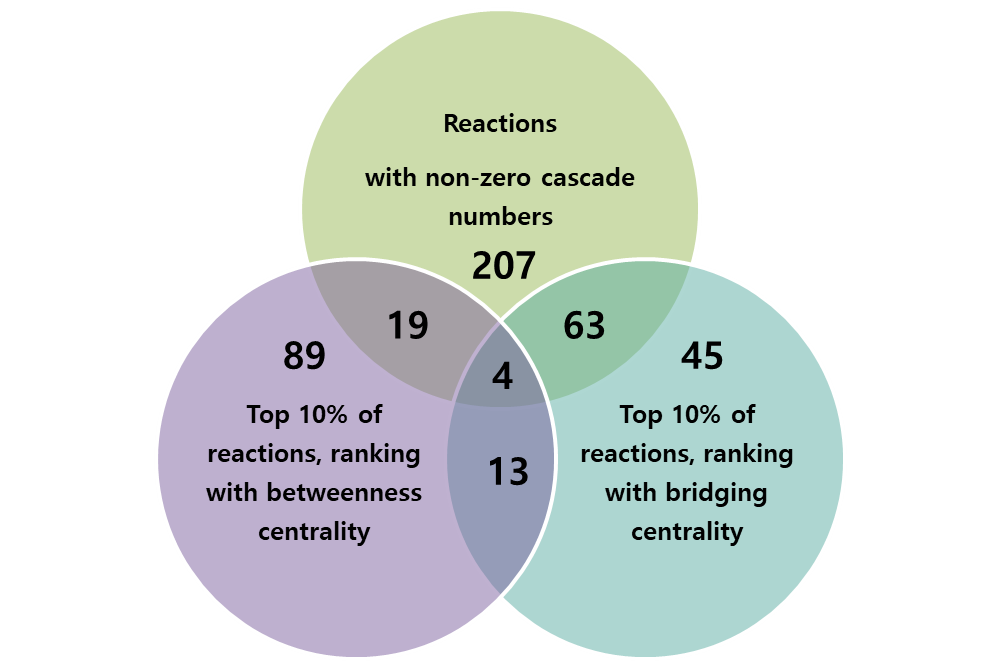
**
